# Early surveillance of rice bakanae disease using deep learning and hyperspectral imaging

**DOI:** 10.1007/s42994-024-00169-1

**Published:** 2024-05-21

**Authors:** Sishi Chen, Xuqi Lu, Hongda Fang, Anand Babu Perumal, Ruyue Li, Lei Feng, Mengcen Wang, Yufei Liu

**Affiliations:** 1 College of Biosystems Engineering and Food Science, Zhejiang University, Hangzhou, 310058 China; 2 State Key Laboratory of Rice Biology and Breeding, Zhejiang University, Hangzhou, 310058 China; 3 Computational Modeling and Nanoscale Processing Unit, National Institute of Food Technology, Entrepreneurship and Management - Thanjavur, Ministry of Food Processing Industries, Thanjavur, 613005 India; 4 College of Environmental and Resource Sciences, Zhejiang University, Hangzhou, 310058 China; 5 Global Education Program for AgriScience Frontiers, Graduate School of Agriculture, Hokkaido University, Sapporo, 060-8589 Japan

**Keywords:** Bakanae disease, Hyperspectral imaging, Deep learning, Early surveillance, Disease monitoring

## Abstract

Bakanae disease, caused by *Fusarium fujikuroi*, poses a significant threat to rice production and has been observed in most rice-growing regions. The disease symptoms caused by different pathogens may vary, including elongated and weak stems, slender and yellow leaves, and dwarfism, as example. Bakanae disease is likely to cause necrosis of diseased seedlings, and it may cause a large area of infection in the field through the transmission of conidia. Therefore, early disease surveillance plays a crucial role in securing rice production. Traditional monitoring methods are both time-consuming and labor-intensive and cannot be broadly applied. In this study, a combination of hyperspectral imaging technology and deep learning algorithms were used to achieve in situ detection of rice seedlings infected with bakanae disease. Phenotypic data were obtained on the 9th, 15th, and 21st day after rice infection to explore the physiological and biochemical performance, which helps to deepen the research on the disease mechanism. Hyperspectral data were obtained over these same periods of infection, and a deep learning model, named Rice Bakanae Disease-Visual Geometry Group (RBD-VGG), was established by leveraging hyperspectral imaging technology and deep learning algorithms. Based on this model, an average accuracy of 92.2% was achieved on the 21st day of infection. It also achieved an accuracy of 79.4% as early as the 9th day. Universal characteristic wavelengths were extracted to increase the feasibility of using portable spectral equipment for field surveillance. Collectively, the model offers an efficient and non-destructive surveillance methodology for monitoring bakanae disease, thereby providing an efficient avenue for disease prevention and control.

**Supplementary Information:**

The online version contains supplementary material available at 10.1007/s42994-024-00169-1.

## Introduction

Rice (*Oryza sativa* L.) is the major crop food in many regions (Li et al. 2022), particularly in Asian countries where rice provides the most dietary caloric supply and a substantial part of the protein intake for the population (Masuda 2019; Muthayya et al. 2014). However, the occurrence of rice diseases can seriously affect rice quality and yield. Bakanae disease, first reported in Japan in 1828 (Ito 1931), is one of the most serious diseases affecting rice production. This disease currently occurs in major rice-growing areas of Asia, Europe, Africa, and North America with an increasing disease incidence (Volante et al. 2017). Significant yield losses caused by bakanae disease can range from 20 to 70% (Gupta et al. 2015; Rood 2004).

The pathogen of bakanae disease was initially named as *Gibberella fujikuroi* (Sawada) Wollenw (Ito 1931), and its anamorph was named as *Fusarium moniliforme* (*F. moniliforme*) (Wilson and Margolis 1982). In recent years, some scholars have classified *F. moniliforme* into different *Fusarium* species, based on morphological, physiological and phylogenetic analysis, and renamed its anamorphs as *Gibberella fujikuroi* species complex (GFSC). GFSC primarily includes three major categories of *F. fujikuroi*, *F. verticillioides*, and *F. proliferatum*, among which *F. fujikuroi* is the main pathogenic fungus (Katoch et al. 2019; Sunani et al. 2020).

After infection, the main substances produced by distinct types of pathogens may vary, and the corresponding symptoms also vary. The typical symptoms of bakanae disease are overgrowth, chlorosis and rotting of slender leaves, crowns and stems (Wulff et al. 2010), and a few are dwarfing or asymptomatic. Postinfection, the internodes of diseased seedlings will elongate abnormally, in comparison to healthy seedlings, resulting in abnormal root development. Some diseased seedlings will wither before transplantation, resulting in empty ears and ultimately affecting yield and seed quality (Desjardins et al. 2000; Zainudin et al. 2008b).

Bakanae infection is a seed-borne disease, the conidium or mycelium within or on the seed surface are important primary sources. Post-germination, the fungi invade the bud sheath, root and root cap and then the mycelium grows between the root and stem (Hossain et al. 2016; Sunani et al. 2020). With the spread by wind or water, conidium produced within the diseased plant can invade healthy seedlings, via wounds, and cause infection, thereby spreading disease within the field. The fungi can also exist in the form of thick-walled hyphae or megaspores in the soil, causing field infections (Saremi and Farrokhi 2004). In addition, during the seed soaking and germination process, the conidium on the infected seeds can spread to the healthy seeds, which then further spreads the fungus.

Recent studies established that *F. fujikuroi* can produce and secrete gibberellic acid (GA_3_), which can stimulate internode elongation (Zainudin et al. 2008a). Accumulation of metabolites, such as fusaric acid (FA) and moniliformin (MON), can increase within infected host plants that can then inhibit chlorophyll synthesis and root growth, which may affect the rate of photosynthesis (Kim et al. 2018; Quazi et al. 2016). Moreover, the pathogenic fungi can also produce other fungal toxins, such as fumonisin B1 (FB1), which pose a threat to the health of humans and livestock (Escriva et al. 2015). Presently, there is little research on the internal physiological changes in rice, postinfection. Further research will contribute to developing an understanding the pathogenic mechanism of the disease.

Once the disease occurs in the rice seedling, within the field, it cannot be overcome; therefore, monitoring and diagnosis of rice seedling disease is of foremost importance. Traditional detection methods for plant diseases typically rely on manual inspection of a crop, by an agronomist, to identify already visible signs of infection. However, this method of monitoring plant health is time-consuming, laborious, and cannot be broadly deployed. Simultaneously, manual detection also relies on obvious symptoms of diseases or stress, making early surveillance of diseases difficult. In addition, the main diagnosis method of plant fungal disease is still molecular technology, such as polymerase chain reaction (PCR) (Sunani et al. 2019), real-time PCR (Amatulli et al. 2012), and loop-mediated isothermal amplification (LAMP) (Zeng et al. 2017). Traditional molecular detection techniques can identify pathogenic fungi and provide feedback on the occurrence of diseases to a certain extent; however, there are disadvantages, including high cost, complex experimental procedures, and time-consuming.

Another constraint is that as the presence of a certain amount of GFSC on seeds, healthy growing rice seedlings can also test positive, so the results obtained by molecular technologies may not offer an accurate disease diagnosis. Moreover, these technologies are destructive and cause significant sample loss. Thus, an alternative, non-destructive technology is needed for early surveillance and diagnosis of rice bakanae disease. Modern optical imaging sensor technologies, such as RGB imaging, multispectral imaging, hyperspectral imaging, and chlorophyll fluorescence, have been widely applied (Lowe et al. 2017) and assessed (Mahlein 2016).

Hyperspectral imaging has shown great potential as a non-invasive and non-destructive tool, and has potential for monitoring of plant diseases. This technology can obtain spatial information of objects and spectral information across hundreds of continuous wavelengths, and provide indicators for early identification of plant diseases, through detection of changes in spectral response caused by disease stress on plants (Wu and Sun 2013). Hyperspectral imaging can accurately, timely and extensively determine the physiological status of crops, detect early disease transmission, and avoid significant crop losses (Terentev et al. 2022).

Hyperspectral imaging has been widely used in plant disease detection of plants, including rice (Zhang et al. 2020), wheat (Guo et al. 2020), cotton (Yan et al. 2021), and squash (Abdulridha et al. 2020), establishing an important potential for plant disease detection. It has also been employed in the field for early surveillance of plant diseases. Zhao et al. (2022) used hyperspectral imaging to obtain data of rice leaves, combined with spectral characteristic wavelengths and image texture features, and proposed a disease level classification method, based on Adaptive weight Immune Particle Swarm Optimization-Extreme Learning Machine (AIPSO-ELM), which further improved the accuracy of rice leaf blast detection. In addition, Terentev et al. (2023) conducted early detection of wheat leaf rust based on hyperspectral imaging; in combination with SVM, the accuracy of early disease detection achieved 97–100% from the fourth day after inoculation. Zhang et al. (2023) proposed a micro-hyperspectral method for the detection of rice fungal disease spores. A microscopic hyperspectral instrument was used to collect hyperspectral data of fungal spores in the enrichment zone, providing innovative ideas for early detection of rice fungal diseases.

The classification or regression analysis of various sensor data typically relies on machine learning (ML). Advances in computing technology have fostered the development of new and powerful deep learning (DL) techniques that demonstrate enormous potential in a wide range of applications (Schmidhuber 2015). Recently, this research trend of DL models has significantly increased, especially for convolutional neural network (CNN) models (Kamilaris and Prenafeta-Boldú 2018; Paoletti et al. 2019). DL models have powerful image-processing capabilities and can automatically extract features through the stacking of convolutional layers, thus occupying a critical position in the field of image-processing research (Too et al. 2019).

Studies have also focused on combining multiple DL and hyperspectral imaging approaches for crop disease detection. Nagasubramanian et al. (2019) achieved high-precision detection of soybean charcoal rot using a 3D deep convolutional neural network (DCNN). Feng et al. (2021) introduced deep transfer learning for detection of rice diseases in different rice varieties, and explored an efficient and cost-effective field detection method for rice diseases among these different varieties.

Unfortunately, when pursuing a larger database and model performance, the network depth continues to deepen, thereby consuming a large amount of hardware resources and runtime. Bulky high-performance hardware may not be suitable for on-site applications in agriculture. Research has shown that, for hyperspectral data, shallow DL models can provide sufficient features to achieve the same outcomes as deep models (Li et al. 2020). Thus, the simple structure and low computational cost of shallow DL models make them a promising system for mobile applications.

In this study, we explored the disease manifestations of infected rice and proposed an early diagnosis method, using hyperspectral imaging with a shallow DL model, to detect rice bakanae disease. The objectives of the study were as follows: (a) to explore the phenotypic trait changes of rice seedlings, over time, after infection with *F. fujikuroi*, (b) to determine the associated spectral regions of rice seedling leaves under the stress of bakanae disease, (c) to classify the infected rice seedlings and select characteristic wavelengths as identifiers for bakanae disease, and (d) to develop a fast and non-destructive early diagnosis method for bakanae disease.

## Results

### Phenotypic traits

**Fig. 1:**
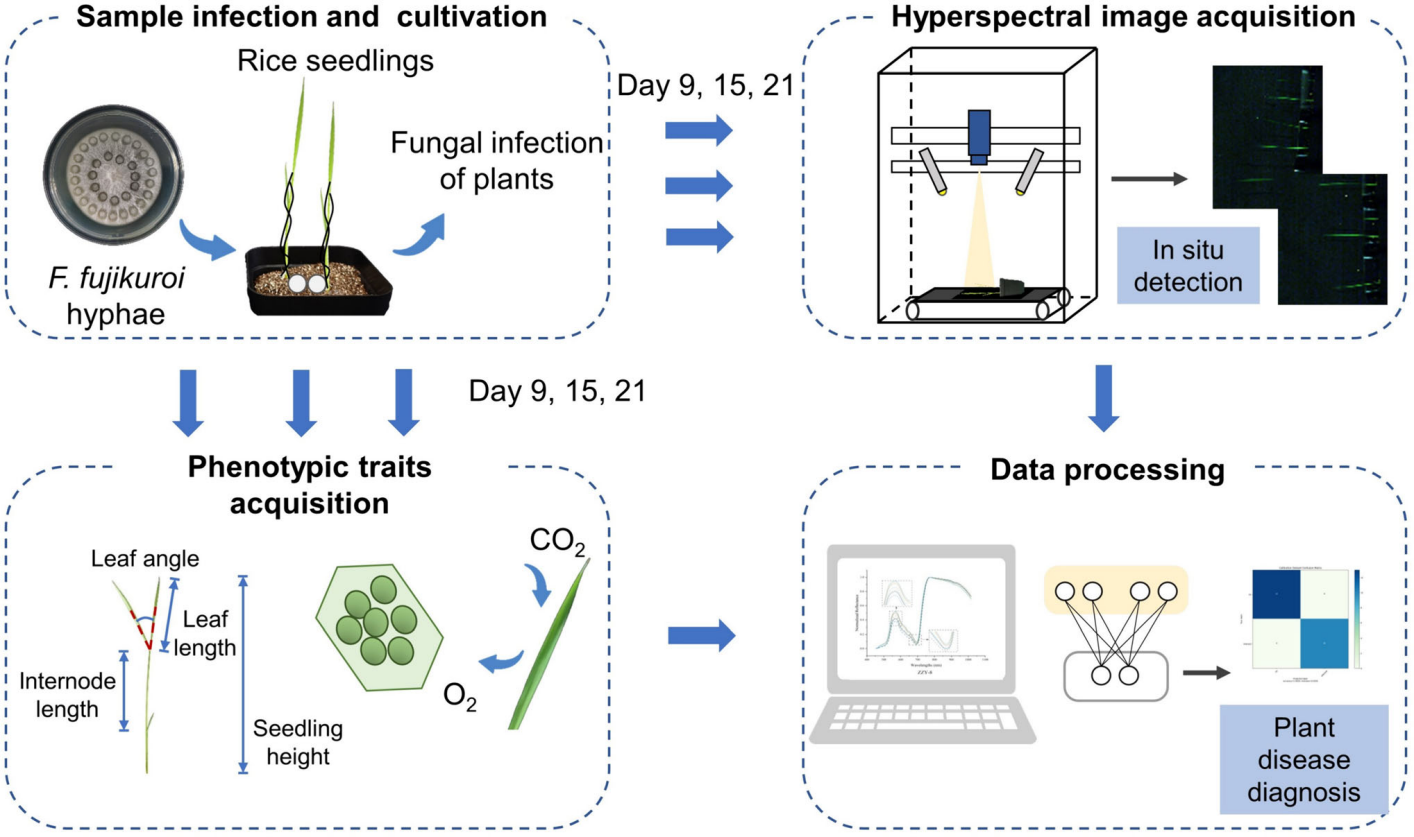
The procedures for data collection and processing

**Fig. 2:**
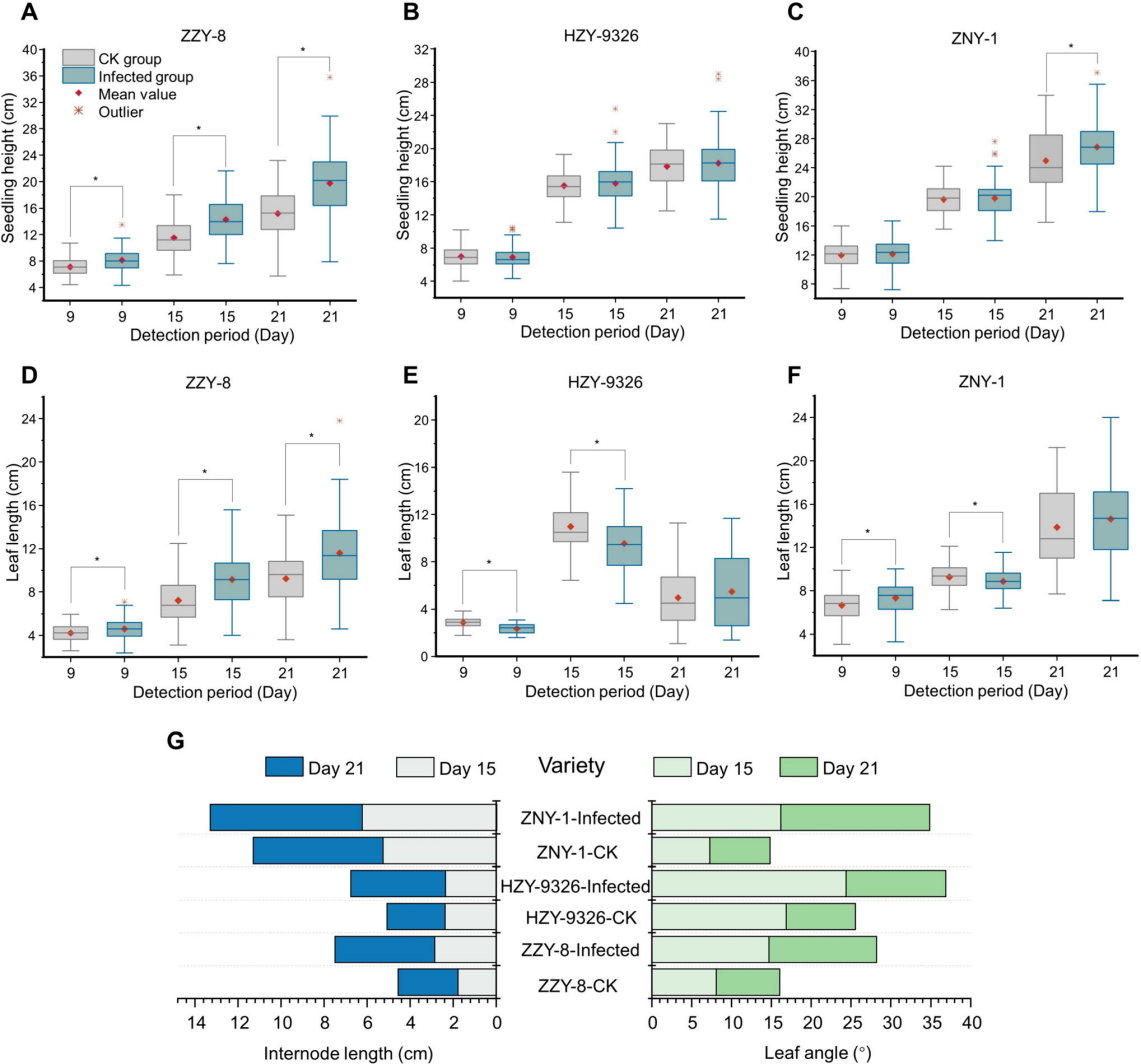
Comparison of morphological traits between infected and healthy seedlings in three periods (* means significant difference with *P* < 0.05): **A** Seedling height of variety ZZY-8. **B** Seedling height of variety HZY-9326. **C** Seedling height of variety ZNY-1. **D** Leaf length of variety ZZY-8. **E** Leaf length of variety HZY-9326. **F** Leaf length of variety ZNY-1. **G** Average internode length and leaf angle of seedling samples. **H** Measured phenotypic characteristics of rice seedlings

The specific process employed in this study is illustrated in Fig. 1. The typical symptoms of bakanae disease are abnormal elongation (Fiyaz et al. 2016), and seedling length data showed that the gap between infected and healthy plants widened with the increase of infection time. Different varieties exhibited varying degrees of susceptibility, with ZZY-8 exhibiting the most significant susceptibility symptoms (*P* < 0.05). As for HZY-9326 and ZNY-1, there were differences in plant height between infected and healthy seedlings, but the overall difference was relatively small (Fig. 2A–C). High outliers existed among infected seedlings of all three varieties, indicating the presence of seedlings with severe infection levels. Similar phenomena were observed in the leaf length section (Fig. 2D–F). Variety ZZY-8 and ZNY-1 showed significant leaf elongation throughout the entire process after inoculation (*P* < 0.05), and the leaf length of infected seedlings was longer than healthy seedlings on the 21st day for HZY-9326. At this stage of infection, the leaves of infected seedlings were elongated by approx. 5–21% compared to healthy seedlings. The internode elongation of infected seedlings was also highly significant, with an elongation amount between 17 and 66% on the 21st day (Fig. 2G). These data indicate that abnormal elongation of internodes is the main reason for plant height elongation.

Infected seedlings also exhibited abnormal growth, with a significant increase in leaf angle (Fig. 2G). The leaf angle of ZNY-1 increased the most, with an increase of 123% on the 15th day and 147% on the 21st day. HZY-9326 increased the least but still caused an enlargement of 44% on both the 15th and 21st day. These results established that during establishment of the bakanae disease, rice seedlings significantly increase horizontally.

**Fig. 3:**
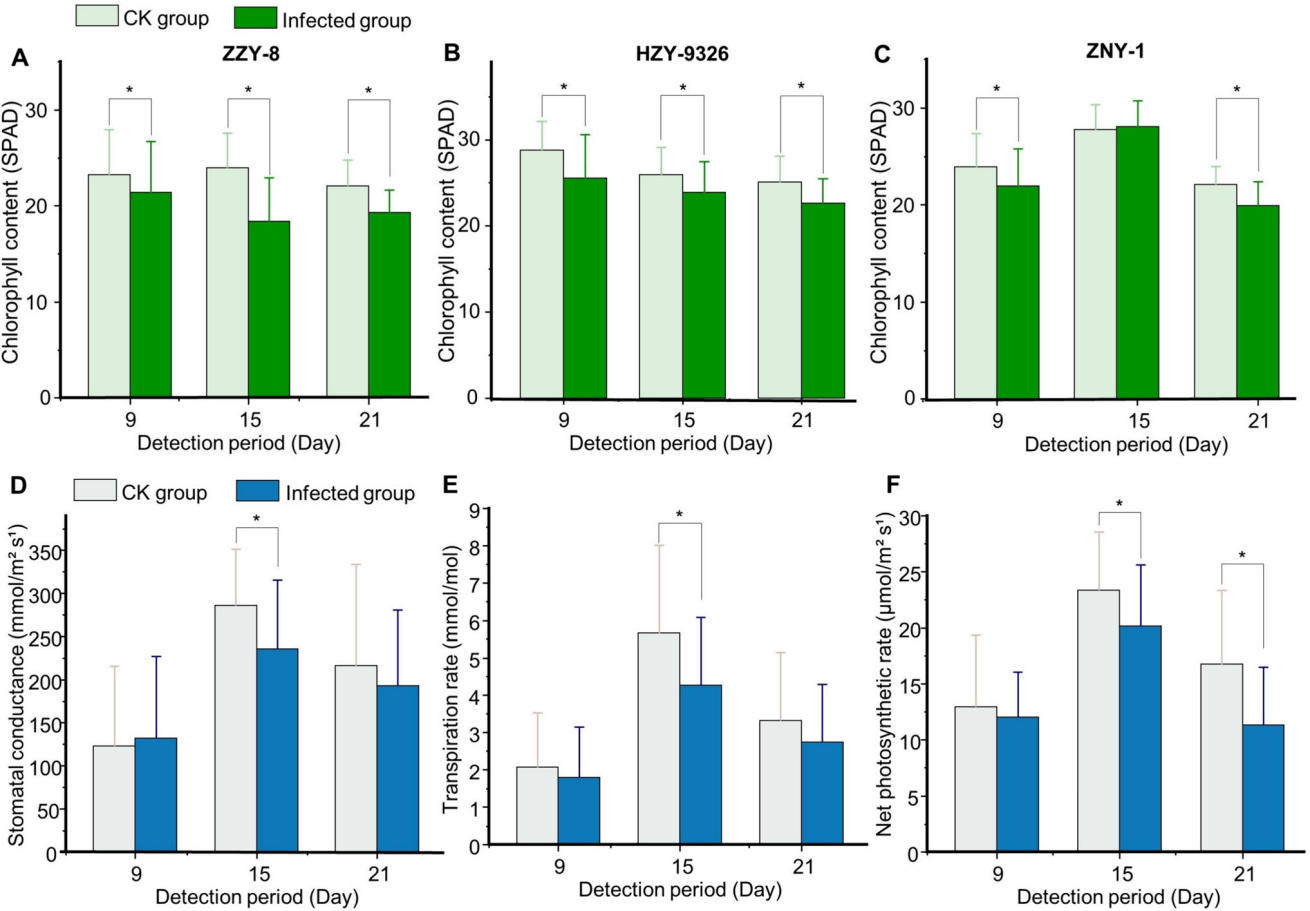
Comparison of chlorophyll content and photosynthetic parameters of rice seedlings in three periods (* means significant difference with *P* < 0.05). **A** Chlorophyll content of variety ZZY-8. **B** Average chlorophyll content of variety HZY-9326. **C** Chlorophyll content of variety ZNY-1. **D** Stomatal conductance of seedling samples. **E** Transpiration rate of seedling samples; **F** Net photosynthetic rate of seedling samples

As shown in Fig. 3A–C, after inoculation with pathogenic fungus, there was a significant trend of decreasing chlorophyll content in seedlings of several rice varieties compared to that in healthy rice seedlings (*P* < 0.05). On the 9th and 21st days, the chlorophyll content of susceptible rice seedlings, of all three varieties, decreased by approx. 10% compared to that of healthy seedlings. During these two periods, there was a consistent degree of chlorophyll damage in by the *F. fujikuroi* products. On the 15th day, the difference in chlorophyll content between infected and healthy seedlings of HZY-9326 and ZNY-1 decreased. This trend was similar to the changes in plant height and leaf length of the infected and healthy groups of these two varieties of rice seedlings. However, continuous infection with pathogenic fungi causes rice seedlings to exhibit symptoms at a later stage.

Fungal infection also affected the photosynthetic performance of rice seedlings (Fig. 3D–F). The stomatal conductance of rice seedlings decreased on the 15th and 21st days after inoculation, thereby affecting the photosynthetic and transpiration rates of these seedlings. Since the latest growing leaves were detected each time, the photosynthetic parameters on the 15th day are higher than those on the 21st day. However, comparing the parameters of the infected and the healthy group, it was clear that, as the disease progressed, the difference in their photosynthetic performance became increasingly significant (*P* < 0.05). When compared to healthy seedlings, the average transpiration rate and average net photosynthetic rate of susceptible seedlings decreased by 18% and 33% on the 21st day, respectively. These changes could seriously impact growth and development of the infected rice seedlings.

### Overview of spectral profiles

**Fig. 4:**
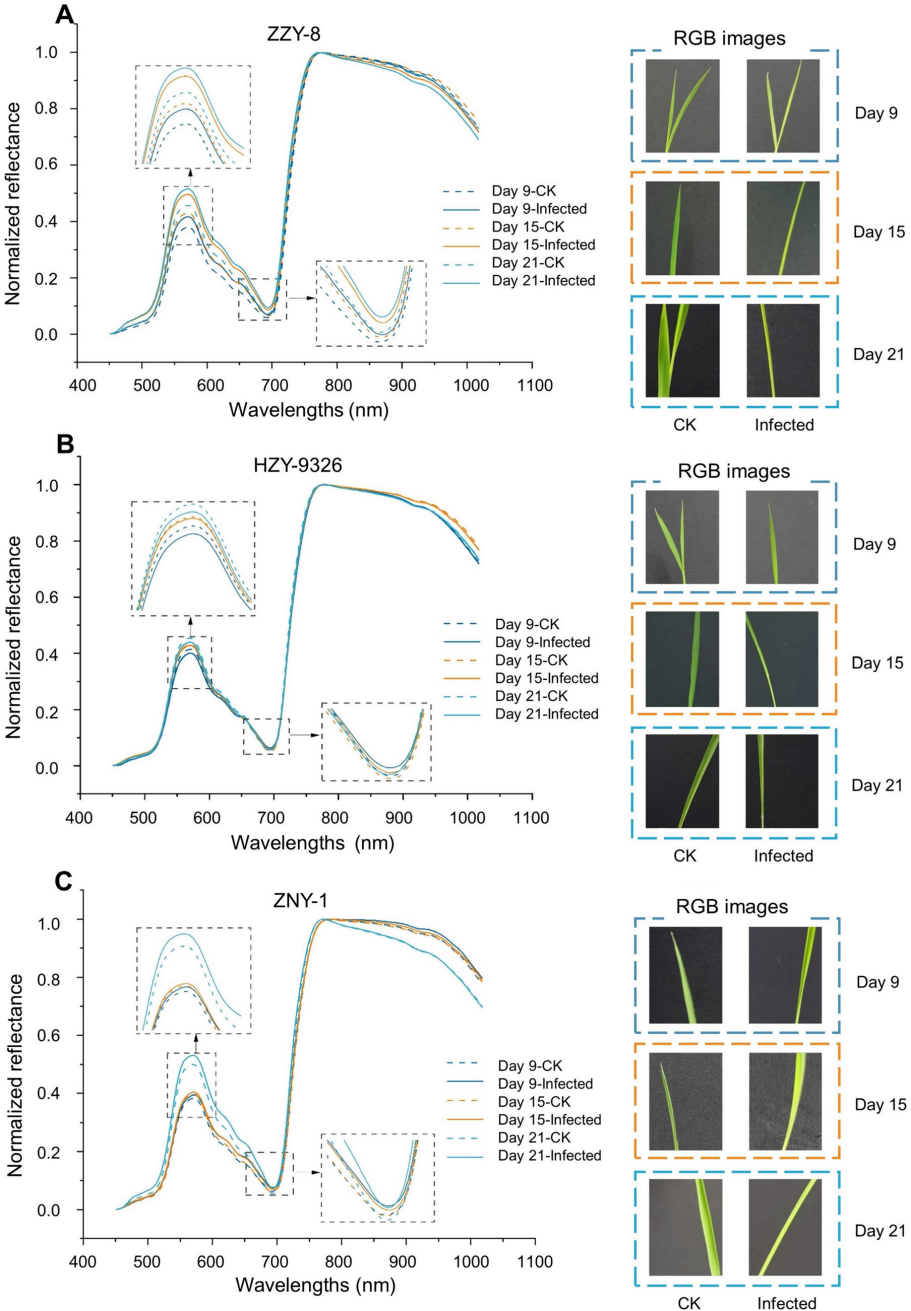
The normalized spectra and RGB images of seedling samples in three periods. **A** Variety ZZY-8; **B** Variety HZY-9326; **C** Variety ZNY-1

To better compare the spectral differences between separate groups, normalization was used to standardize the spectral data. A total spectral data range between 450 and 1017 nm is presented in Fig. 4, and there was an obvious absorption peak and a valley in the visible region. Regarding the absorption peak at 570 nm, for most varieties, the peak values for infected rice seedlings, at each stage, were higher than those of the healthy seedlings. Generally, the differences in peak values between the two groups of samples on the 21st day were the largest. In the visible region, the wavelength ranges of green, yellow-green, and yellow are 510–560, 560–580, and 580–595 nm, respectively. The 570 nm wavelength is located in the malabsorption area of various pigments and has been used to calculate the index of chlorophyll (Gitelson et al. 2006), xanthophyll (Letts et al. 2008), and other pigments (Sanchez et al. 2022). In the range of 570–700 nm, the reflectance of infected rice seedlings was higher than for healthy seedlings, indicating that the infected seedlings reflected more yellow, confirming the symptoms of chlorosis caused by bakanae disease. This may be due to the differences in pigment content among varieties, the reflectance of variety HZY-0326, at 570 nm, was different from that of the other varieties, and the difference between the infected and healthy groups was not significant.

An absorption valley was observed at 693 nm, which is located near the red edge position of the vegetation spectrum that is sensitive to biochemical changes and is highly sensitive to chlorophyll content (Gitelson and Merzlyak 2004; Vogelmann and Moss 1993). All samples from the tested varieties showed the same trend, with infected seedlings exhibiting higher reflectance than healthy seedlings during the same period. These results indicated a decrease in chlorophyll content, which was consistent with the chlorophyll content detection results. Variety ZZY-8 exhibited the most obvious pattern of overall differences, reflecting the deepest degree of infection.

### Classification based on full wavelengths

The classification results based on the entire 222 spectral bands are shown in Table 1. Three classification models were used to compare the effects. These results showed that the model classification became more accurate as the infection time increased. All three models reached an average accuracy of over 88% on the 21st day, verifying the feasibility of spectral detection of bakanae disease. Owing to the short infection time and unclear degree of infection, the accuracy on the 9th day was not high. The SVM model reached an average accuracy of 68.9% on the 9th day. The two DL models improved accuracy by 3.6% and 10.5%, respectively, in the same period. The Rice Bakanae Disease-VGG (RBD-VGG) model performed the best among the three models, reaching an accuracy of over 80% in two varieties on the 9th day, and highly improved the accuracy of early surveillance. Moreover, the accuracy of the calibration set for each period and variety reached 100%. On the 21st day, the RBD-VGG model achieved an average accuracy of more than 90%, with an accuracy of 95.5% for variety HZY-9326. In terms of the average accuracy, during the different periods, the RBD-VGG model was improved by 10.5%, 9.2% and 3.6% compared to the SVM model, and by 6.9%, 1.0% and 3.5% compared to the CNN model, respectively. The comparison results showed that the DL model had a better classification performance than ML in the detection of bakanae disease, based on spectral data. Among them, the improved RBD-VGG model in this study performed better than the CNN model and achieved early high-precision classification.

**Table 1:** Classification accuracy based on full wavelengths

Model	Day	Variety	Accuracy (%)			Average accuracy (%)	Parameters
			Cal	Val	Pre	Pre	
SVM	9	ZZY-8	63.9	75.7	62.2	68.9	10^–7^, 10^7^
		HZY-9326	82.6	69.6	52.2		10^–3^, 10^7^
		ZNY-1	96.2	88.5	92.3		10^–3^, 10^7^
	15	ZZY-8	97.4	82.5	77.5	76.7	10^–4^, 10^8^
		HZY-9326	61.1	80.0	68.0		10^–1^, 10^2^
		ZNY-1	97.4	96.2	84.6		10^–2^, 10^5^
	21	ZZY-8	98.3	94.9	92.3	88.6	10^–4^, 10^6^
		HZY-9326	93.9	90.9	90.9		10^–2^, 10^5^
		ZNY-1	100	100	82.6		10^–3^, 10^7^
CNN	9	ZZY-8	95.4	91.9	64.9	72.5	1500
		HZY-9326	98.6	95.7	56.5		1500
		ZNY-1	100	96.2	96.2		2000
	15	ZZY-8	94.9	90.0	90.0	84.9	1500
		HZY-9326	84.7	96.0	84.0		1500
		ZNY-1	100	100	80.8		1500
	21	ZZY-8	90.4	98.7	97.2	88.7	2000
		HZY-9326	80.3	90.9	86.4		2000
		ZNY-1	92.7	91.3	82.6		1500
RBD-VGG	9	ZZY-8	100	70.3	70.3	79.4	2000
		HZY-9326	100	73.9	82.6		2000
		ZNY-1	100	92.3	84.6		2000
	15	ZZY-8	100	67.5	85.0	85.9	2000
		HZY-9326	100	80	88		1500
		ZNY-1	100	96.2	84.6		1500
	21	ZZY-8	100	82.1	89.7	92.2	2000
		HZY-9326	87.9	95.5	95.5		1500
		ZNY-1	100	82.6	91.3		2000

Parameters for SVM model are gamma and c (regularization parameter); for CNN and RBD-VGG model is epoch

### Characteristic wavelengths selected

**Fig. 5:**
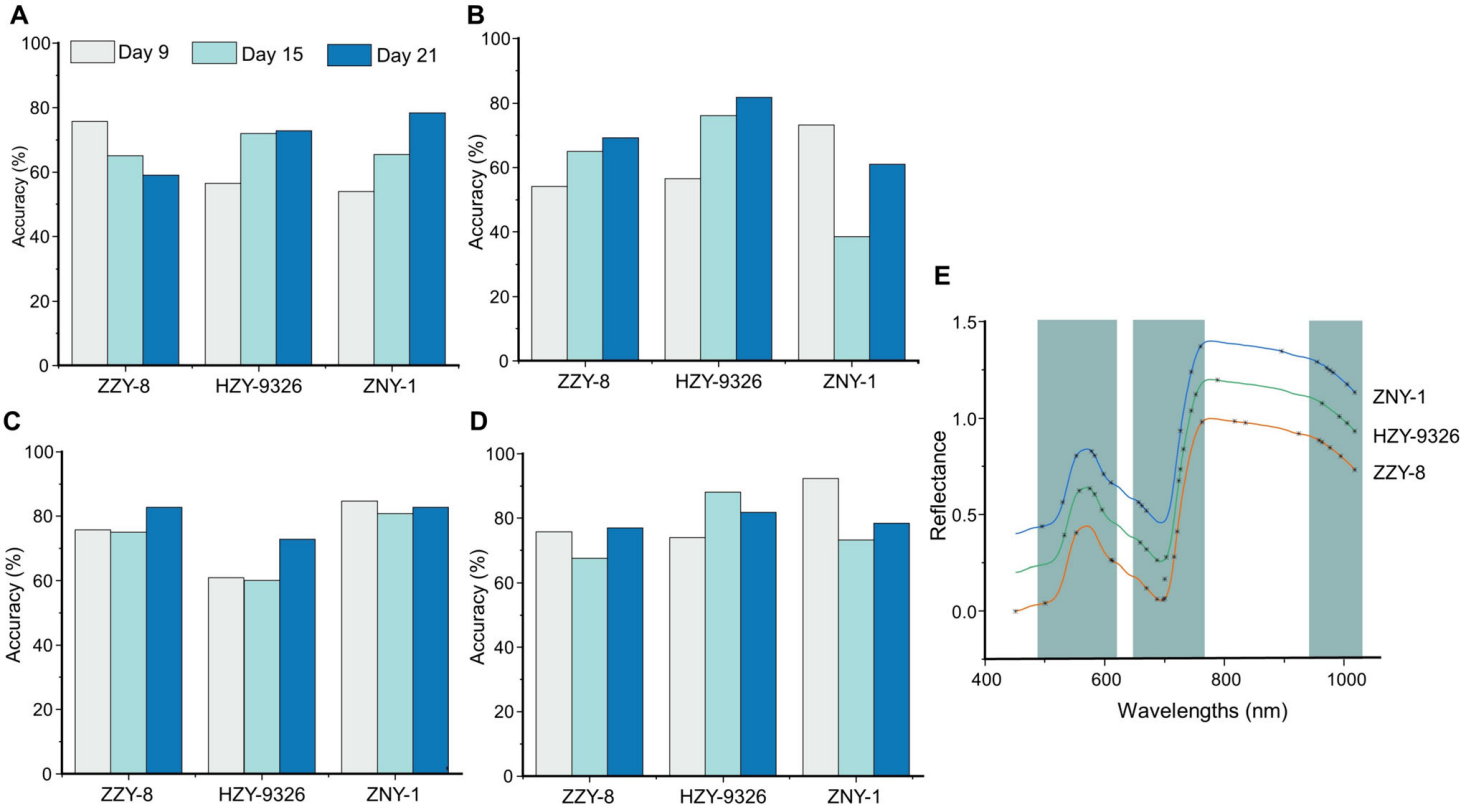
Classification accuracy of prediction set based on SPA and CARS wavelength extraction algorithms. **A** Five characteristic wavelengths extracted by SPA. **B** Five characteristic wavelengths extracted by CARS. **C** Ten characteristic wavelengths extracted by SPA. **D** Ten characteristic wavelengths extracted by CARS. **E** Characteristic wavelengths of three rice varieties extracted by SPA and CARS algorithms

To further extract useful spectral information and facilitate future instrument simplification, the SPA and CARS algorithms were used for characteristic wavelength selection and RBD-VGG was used for further detection. The two algorithms were used to extract five and ten characteristic wavelengths for each variety, respectively (Table S1). The accuracy of the prediction set based on characteristic wavelengths is shown in Fig. 5A–D. When the number of wavelengths were simplified to five, the classification accuracy of the model generally was between 53 and 82%. The accuracy could generally reach the highest on the 21st day, and using the CARS algorithm, 81.82% accuracy was achieved on the 21st day for the variety HZY-9326. When the number of wavelengths reached 10, the classification accuracy of the model significantly improved to 60–93%.

The performance of the feature bands extracted by the two algorithms varied. The wavelengths extracted using the SPA algorithm had better classification performance for the variety ZZY-8 and ZNY-1, whereas the wavelengths extracted using the CARS algorithm had better classification performance for the variety HZY-9326. Therefore, in this study, we combined the characteristic wavelengths extracted by the two algorithms; and the specific wavelength positions are shown in Fig. 5E. Here, we observed that the characteristic wavelengths were concentrated between 495 and 610 nm, 656 and 760 nm, and 955 and 1017 nm for different varieties of rice seedlings, with a high degree of overlap. This indicates that these bands mainly reflect the impact of bakanae disease on rice seedlings, as the 495–610 nm region reflects leaf chlorosis and the 656–760 nm region reflects a decrease in chlorophyll content.

### Classification based on characteristic wavelengths

The detailed classification accuracy, based on 20 characteristic wavelengths selected by SPA and CARS algorithms, is presented in Table S2. Due to the simplification of spectral information, the training epochs were all 1500. The results showed that the accuracy close to the full wavelength could be achieved using only 20 wavelength classification after feature selection. Compared with the results based on full wavelengths, the prediction accuracy for variety ZZY-8 and ZNY-1 was improved by 10.8% and 3.9% on the 9th day, respectively. The accuracy for variety HZY-9326 on the 9th day was relatively low, but the accuracy on the 15th day was improved to 88% on both the validation set and prediction set. The average accuracy based on the characteristic wavelengths of the 21st day was relatively lower than that based on the full wavelengths. However, the prediction accuracy based on the characteristic wavelengths for ZNY-1 on the 21st day was improved by 8.7%, and the results were satisfactory for all three datasets.

**Fig. 6:**
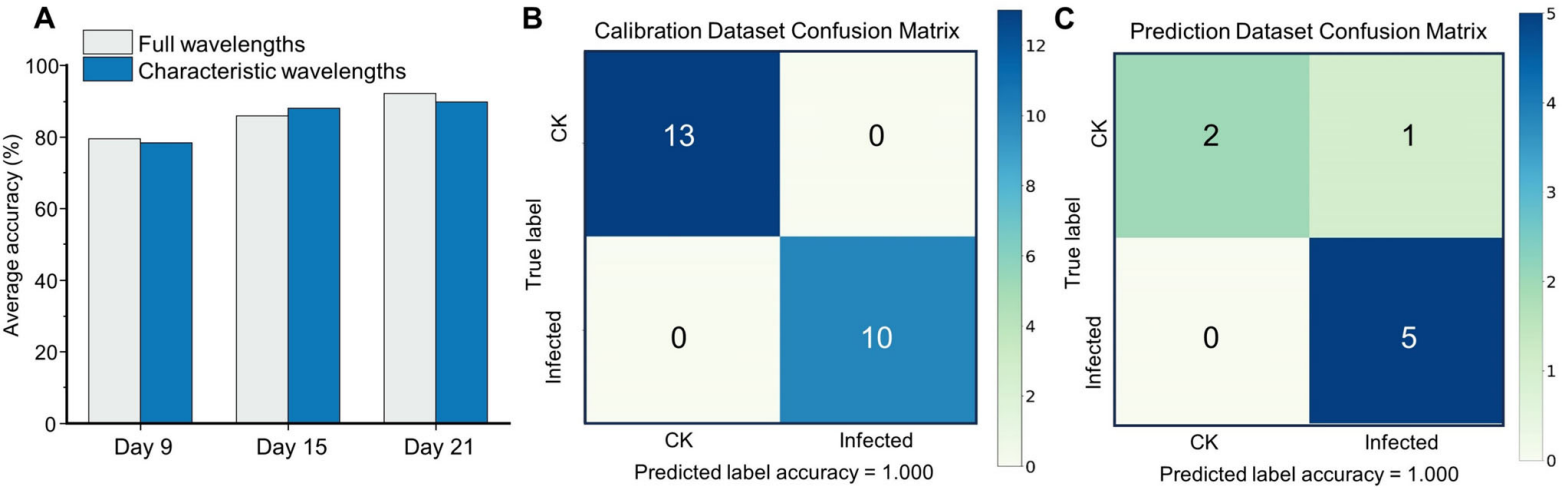
Detection results based on different datasets. **A** Average accuracy of prediction set based on full wavelengths and characteristic wavelengths in the three periods. **B** Confusion matrix on calibration set of two different varieties of rice seedlings, based on the characteristic wavelengths recommended. **C** Confusion matrix on prediction set of two different varieties of rice seedlings, based on the characteristic wavelengths recommended

Overall, the average accuracy of the prediction set, based on full wavelengths and characteristic wavelengths, is shown in Fig. 6A; the accuracy was improved on the 15th day and slightly lower in the other two periods, but was still deemed to be satisfactory. These results indicated that removing a certain amount of redundant information may enhance the learning ability of the model and indicated the feature extraction ability of the model proposed in this study. The almost identical accuracy verifies the feasibility of collecting a small number of wavelengths for the surveillance of bakanae disease.

## Discussion

As shown in Fig. 1, in this study, phenotype data and hyperspectral image data were obtained on the 9th, 15th, and 21st day of rice seedling growth. By constructing a shallow VGG model and combining spectral data, early detection of rice blast disease was achieved. In general, *F. fujikuroi* infection primarily led to various morphological changes in the rice seedlings. From a vertical perspective, seedling height, leaf length, and internodes of rice seedlings would increase. The internodes were elongated most significantly, reaching a maximum of 66%. From a horizontal perspective, the leaf angle of the rice seedlings would also expand, with an average expansion value of 86.7% on the 21st day. Overall, the degree of morphological changes in some samples did not increase over time, whereas morphological changes in some severely affected samples continued to expand and in some cases, the seedlings even withered.

Morphological differences caused by fungal infection resulted in abnormal plant-type proportions in rice seedlings, with very weak stems that were prone to collapse, thus compromising further normal growth. These symptoms were mainly related to the production of the gibberellin, GA_3_. In monocotyledons, stems composed of nodes and internodes affect the plant height and productivity. Here, gibberellins stimulate internode elongation by activating cell division and cell elongation (Nagai et al. 2020). Our findings indicated that symptoms of some infected seedlings did not show significant differences, compared to the healthy seedlings, which indicated that the disease could not be accurately visually observed at the early stage. Therefore, the use of detection technology is needed.

Infection with pathogenic fungi can also lead to some changes within the plant. Such symptom may be caused by an increase in the accumulation of metabolic products from the *Fujikura* fungus (Quazi et al. 2016). Destruction of chlorophyll structure and the reduction in chlorophyll content could lead to significant changes in photosynthetic activity. Our findings indicated that leaf yellowing, caused by the disease, was due to a decrease in chlorophyll content. Further, we established that, with the passage of susceptible time, stomatal conductance of rice seedling leaves significantly decreased, during the last two surveillance periods, causing a decrease in the transpiration and photosynthetic rates. This change in photosynthetic activity may be one of the contributors to the decrease in rice yield.

The classification results indicated that hyperspectral imaging technology could achieve early and accurate surveillance of bakanae disease. Currently, only a few studies have focused on non-destructive detection and diagnosis of bakanae disease. Kim et al. used unmanned aerial vehicle (UAV) RGB images to automatically detect rice seedling disease, during the tillering stage, in the field (Kim et al. 2023). YOLOv3 and RestNETV2101 network models were used to detect infected rice plants and classify the number of infected stems, respectively. Their classification accuracy of the number of infected stems in susceptible plants reached 80.36%. Chung et al. established a detection model for seedlings infected with bakanae disease based on flat-panel scanners and an SVM algorithm, achieving detection in 3-week-old seedling detection (Chung et al. 2016). The average detection accuracy for seedling infection was 87.9%.

In this study, based on the hyperspectral imaging technology and an improved DL model (RBD-VGG), we achieved an average detection accuracy of 92% for seedlings infected for 21 days, with the variety HZY-9326 reaching 95.5%. Moreover, the RBD-VGG method could detect infection earlier in seedlings, after 9 days of infection, with an average accuracy of 79.4%. Therefore, the RBD-VGG method offers advanced detection characteristics.

Earlier studies have shown that combining hyperspectral imaging with drones for large-scale field experiments is more feasible. A comparison of accuracies revealed that DL was more accurate in processing spectral information than ML. Moreover, the predictive accuracy of the RBD-VGG model was significantly higher than that of the SVM model, and its performance was better than that of the CNN model. Hence, the RGB-VGG model can better focus on individual features than the CNN with the ATT block. Meanwhile, high-precision classification results can be achieved based on the 20 characteristic wavelengths obtained by combining the CARS and SPA algorithms. The characteristic wavelengths of different varieties of rice seedlings have a high degree of overlap. Considering all varieties, the recommended characteristic band regions and wavelengths, derived from this study, are shown in Table 2. To verify the rationality of the characteristic wavelength, 31 spectral data of two other different varieties of rice seedlings were obtained, on the 21st day of infection (Table S3). Because of the limited data, the SVM model was used for training, and the confusion matrix was obtained, as shown in Fig. 6B, C, reaching a prediction accuracy of 87.5%. These results validated the universality of the feature wavelengths proposed in this study and also demonstrated the feasibility of using convenient spectral instruments with small wavelengths for rapid detection.

**Table 2:** The recommended characteristic band regions and characteristic wavelengths

Characteristic band regions (nm)	Characteristic wavelengths (nm)
495–610, 656–760, 955–1017	530, 553, 575, 583, 698, 611, 659, 669, 687, 700, 721, 726, 744, 760, 955, 963, 976, 994, 1005, 1017

Hyperspectral imaging technology has a wide range of applications, which can be combined with drones for large-scale field surveillance and can also be used for portable detection in the field. Since the spectral data in this study were obtained, in situ, through a non-destructive method, this satisfactory detection accuracy also demonstrates the potential of using hyperspectral methods for rapid, in situ, detection. Further exploration in field detection of the improved model developed in this study, combined with hyperspectral technology, remains to be conducted in the future.

In conclusion, in this study, we established that the RBD-VGG model can achieve accurate disease detection, based on hyperspectral data, and the network can better focus on highly responsive wavelengths compared to the CNN model. The lightweight architecture of the model has wide applicability, low equipment requirements, and is suitable for on-site use. Furthermore, the characteristic wavelengths of rice seedlings that were highly responsive to the disease were extracted, and their universality was verified. In the future, portable spectral instruments can be combined to achieve rapid detection based on a small number of wavelengths, thereby reducing the instrument costs. Collectively, these findings indicate that hyperspectral imaging and DL have advantages in the rapid and non-destructive surveillance of rice bakanae disease in the early stages, making them potential candidates for monitoring rice seedling growth.

## Materials and methods

### Experimental materials and design

Three varieties of rice seeds, including Zhenuoyou-1 (ZNY-1), Zhongzheyou-8 (ZZY-8) and Huazhongyou-9326 (HZY-9326), were purchased online. Two varieties of seeds, including Jiaheyou-5 (JHY-5) and Xiushui-121 (XS-121), were provided by the College of Agriculture and Biotechnology, Zhejiang University, China. Considering the universality of the method, the selected varieties include japonica rice (HZY-9326, JHY-5, XS-121), glutinous rice (ZNY-1), and indica rice varieties (ZZY-8). Seeds were planted from April to June, 2023. A total of 600 seeds were planted and the specific number of different rice seedling samples is shown in Table S3 and the healthy seedlings were planted as CK group. Hyperspectral data of ZNY-1, ZZY-8 and HZY-9326 were used for model building and data of JHY-5 and XS-121 were used for model validation. The cultivation medium was vermiculite, which was sterilized and disinfected using a high-pressure sterilizer before planting. *F. fujikuroi* was cultivated for about a week by the College of Agriculture and Biotechnology, Zhejiang University, China. Pathogenic fungus, on culture medium, was subsequently made into tablets and put on vermiculite in every seedling pot of infected samples. Rice seeds were disinfected with a hypochlorous acid solution and germinated for 2 days in the dark. Then, the germinated seeds where placed in the pot, and then covered with a shallow vermiculite layer. The samples were grown in an incubator for 3 weeks, with environmental conditions set at 12 h light/28 °C, 12 h dark/20 °C, and a relative humidity of 80%. Due to limitations in rice growth, data collection began on the 9th day, when the shape and size were suitable for accurate measurement. The research focused on early detection, so the final detection period was the 21st day of growth (3 weeks of age), and testing was performed in stages. To comprehensively explore the impact of rice bakanae disease, phenotypic data, chlorophyll content, and photosynthesis parameters, and the hyperspectral data were obtained on the 9th, 15th, and 21st day of rice growth. Among them, the full period data of the first three types of rice seedlings were obtained for the main research, and the 21st day hyperspectral data of the last two types of rice seedlings were obtained for algorithm validation. The specific process of the study is shown in Fig. 1.

### Hyperspectral image acquisition

A visible/near-infrared hyperspectral imaging (Vis/NIR HIS) system was used to capture hyperspectral images from the rice seedlings with a spectral range of 414–1017 nm, producing a total of 237 spectral bands. The system consisted of an imaging spectrograph (ImSpector V10E, Spectral Imaging Ltd., Oulu, Finland) with a spectral resolution of 2.8 nm, a sCMOS camera (Zyla-4.2P, Andor Technology, UK), a high-quality lens (Schneider Kreuznach Xenoplan 1.4/17-0903), 2150-W tungsten halogen lamps (Fiber-Lite DC950 Illuminator, Dolan Jenner Industries Inc., Boxborough, MA, USA), a conveyer operated by a stepper motor (IRCP0076, Isuzu Optics Corp., Taiwan, China) for line scan imaging and a data acquisition software (Xenics N17E, Isuzu Optics Corp., Taiwan, China).

To achieve in situ detection and data acquisition in a dynamic timeline, rice seedlings were always located in the seedling pot during hyperspectral scanning. Pots were tilted and placed so that the seedlings are parallel to the track, and a comprehensive image of the leaves' surface was obtained as much as possible. In the pre-experiment, images of both sides of rice leaves were obtained by flipping the seedling pot, and the spectral differences were analyzed to be small (Fig. S1), verifying that the placement method did not impact the obtained hyperspectral data. Multiple seedling samples were placed on a black plate so the system could perform batch detection. Approximately, 1300 hyperspectral images of rice seedlings were obtained during three experimental stages. The hyperspectral images were captured in a dark room to minimize interference by ambient light. The original images were further corrected using a white (hyperspectral image of a white Teflon tile with a reflectance of approximately 99%) and a dark (hyperspectral image of a black cloth with a reflectance close to 0) reference image to eliminate interference from dark current and other factors according to Eq. 1 (Tahmasbian et al. 2018):

1 $$R = \frac{R_0 - D}{{W - D}}$$

where *R*_0_ is the uncorrected spectral reflectance, *D* is the reflectance of the dark image and *W* is the reflectance of the white Teflon tile.

### Spectra extraction and preprocessing

When obtaining data each time, the latest leaf of each rice seedling was uniformly selected, and images of each rice seedling were cut out in ENVI software. Due to the large difference in reflectance spectra between the leaves and the black background, the leaves were isolated from the background by a threshold segmentation algorithm based on the spectral reflectance of the 210th band. Due to strong noise interference at the beginning of the band, this research only studied the average spectrum of 448–1017 nm. Due to the presence of noise, the original spectral curve exhibited uneven fluctuations and required spectral preprocessing. Due to the good smoothing effect, the Savitzky–Golay filter was used for spectral preprocessing in this study. Savitzky–Golay filter was an improved average recursive filtering method that has the characteristics of simple and fast operation and is a widely used spectral signal processing technology (Zhu et al. 2017). In this study, a Savitzky–Golay filter was chosen to smooth the curve and filter out the noise. The Savitzky–Golay filter was performed based on the signal module in the SciPy library, where the parameter window_Length = 11, polyorder = 2. The min–max normalization shown in Eq. 2 (Chu et al. 2022; Sheela and Deepa 2013) was used to preprocess the average spectrum, which could eliminate deviations between data and improve the classification accuracy of the model. Each variety of seedling data on a single period was divided into a calibration set, a validation set and a prediction set according to a ratio of 3:1:1. The specific data volume of each dataset within each detection stage is shown in Table 3. Due to the withering of some seedlings after being infected, the data volume on the 21st day was decreased to a certain extent:

2 $$R_{\text{n}} = \frac{{R - R_{\min } }}{{R_{\max } - R_{\min } }}$$

where *R*_n_ means the normalized spectral reflectance, *R* means the actual spectral reflectance, *R*_min_ means minimum spectral reflectance, and *R*_max_ means the maximum spectral reflectance.

**Table 3:** Specific data volume of each dataset within each detection stage

Stage	Dataset		
	Training set	Validation set	Prediction set
9th day	268	89	89
15th day	271	90	90
21st day	250	84	84

### Phenotypic traits acquisition

Different sensors were utilized to further explore changes in the phenotypic traits of rice seedlings after infection. The seedling height, internode length, and leaf length of each rice plant were measured manually by an agronomist, and the average of three replicates was taken. Seedlings images were taken and the leaf angle was measured using the software ImageJ (National Institutes of Health, Germany). To compare the recent symptoms of the disease, the newly grown internodes, leaves, and leaf angle on the day of detection were selected for measurement. The specific measurement features are shown in Fig. 1. Due to chlorosis being one of the typical symptoms of rice bakanae disease, the SPAD chlorophyll meter (SPAD-502, Konica Minolta, Japan) was used to simultaneously obtain the chlorophyll content of healthy and infected rice leaves. During measurement, the SPAD chlorophyll meter was used to repeatedly obtain data twice, at different sites on the leaf, and the average value taken. Li-6800 portable photosynthesis system (Li-Cor Biosciences, Lincoln, NE, USA) was used to obtain photosynthesis parameters, including net photosynthetic rate, transpiration rate, and stomatal conductance. Similarly, the latest growing leaves were selected for measurement of chlorophyll content and photosynthesis parameters.

### Chemometrics analysis

**Fig. 7:**
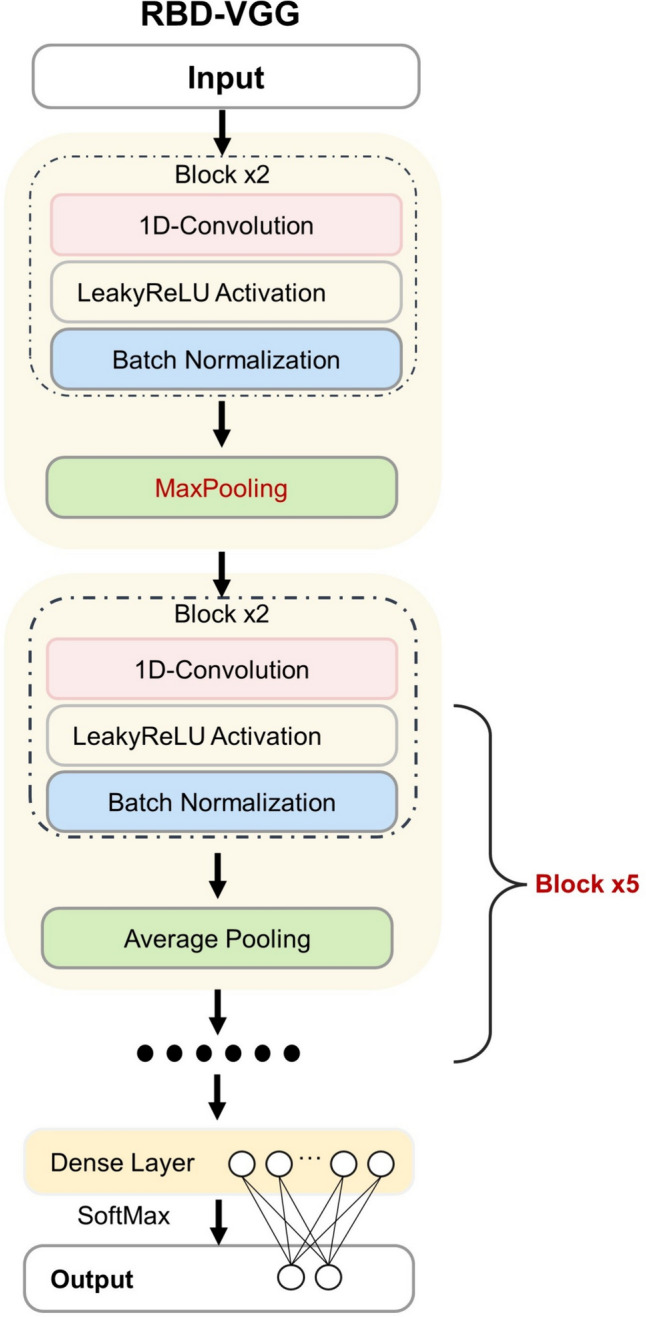
Architecture of the RBD-VGG

In this research, three popular AI models including a support vector machine (SVM) model and two DL models were used to achieve rapid and accurate classification based on spectral data. SVM is an important classification and regression method in the field of pattern recognition and ML has been widely applied to many real-world problems (Yang et al. 2013). The main advantage of SVM is that it can construct a decision rule that can be well generalized in high-dimensional feature spaces, thereby achieving high accuracy (Aburomman and Reaz 2017). The main parameters of the SVM model used in this study are the gamma and regularization parameter. The model can automatically find the optimal parameter combination based on its performance on the calibration set and validation set, thereby achieving the best classification effect. CNN is a widely used DL algorithm that has been applied in various fields. A custom block with an attention mechanism named attention block (ATT block) was introduced in CNN, which allowed the network to pay more attention to informative parts of input data. Complex frameworks can lead to overfitting, so the shallow CNN network (Chu et al. 2022) used in this study consists of one convolutional block consisting of a batch normalization layer, a 1D-Conv layer, an exponential linear unit (ELU) function, and a MaxPooling layer. Subsequently, there are three dense layers and dropout technology is adopted. The number of kernels in the 1D-Conv layer was set as 16, with a kernel size of 3, stride of 1. The pool size and stride of MaxPooling were set as 2. The number of neurons in each dense layer was 512, 128 and 2. After adjustment, the training parameters were as follows: batch size = 32; the initial learning rate (lr) = 0.001, decreasing to 1/10 of the original for every 250 epochs; the epoch was adjusted based on the specific effect. The Visual Geometry Group (VGG) architecture consists of similar layer blocks. Each VGG block contains two convolutional layers and one pooling layer. VGG has a small convolutional kernel and a small pooling kernel, which not only ensures visual perception but also reduces the parameters of the convolutional layer and has strong learning ability for features. A shallow DL model named RBD-VGG based on SpectraVGG architecture (Blazhko et al. 2021) was attempted in this study. This model has a lightweight architecture with fewer weights, and low computational costs and performs well on multiple spectral datasets. Figure 7 shows the architecture of RBD-VGG, the network consists of six blocks and then there is the dropout layer (with a rate of 0.5) and the fully connected (dense) layer for classification. In this study, more VGG blocks were added to enhance the model's ability to capture details while ensuring a balance between network depth and computational resources. The filter size is 3. The first VGG block has 12 filters, and the following blocks have 24, 48, 96, 192, and 384 filters, respectively. Each VGG block has two 1D-convolution (1D-Conv) layers. After each Conv layer, a Leaky rectified linear unit (LeakyReLu) [formula (1)] function and batch normalization layer are added. In the first block, the pooling layer was changed to MaxPooling with a pool size of 8 and a step size of 4, which could more easily capture changes in images, resulting in greater local information differences and accelerating convergence. In the subsequent blocks, the AveragePooling layer is used to reduce overfitting and ensure model robustness, with a fixed pool size and step size of 2. Equations 3 and 4 demonstrate the calculation principles of the two pooling layers. The training parameters were the same as shallow CNN:

3 $$F\left( x \right) = \max \left( {\alpha x,x} \right)$$

where *x* is the value input to the activation function; *α* is a small positive coefficient used to input the multiplication factor when *x* is less than 0:

4 $${\text{Output}}\,\left( {i,j} \right) = \max_{m,n} \left( {{\text{Input}}\left[ {i \times s + m, j \times s + n} \right]} \right)$$

where Output [*i*, *j*] is the value of the output feature map at position (*i*, *j*); Input [*i* × *s* + *m*, *j* × *s* + *n*] is the value of the input feature map at position (*i* × *s* + *m*, *j* × *s* + *n*), where *s* is the stride of the pooling window; *m* and *n* traverse the elements within the pooling window:

5 $${\text{Output}}\,\left[ {i,j} \right] = \frac{1}{N}\mathop \sum \limits_{m = 1}^M \mathop \sum \limits_{n = 1}^N {\text{Input}}\left[ {i \times s + m,j \times s + n} \right]$$

where Output [*i*, *j*] is the value of the output feature map at position (*i*, *j*); *N* is the total number of elements in the pooled window; Input [*i* × *s* + *m*, *j* × *s* + *n*] is the value of the input feature map at position (*i* × *s* + *m*, *j* × *s* + *n*), where *s* is the stride of the pooling window; *m* and *n* traverse the elements within the pooling window; *M* and *N* are the height and width of the pooling window.

The models performed classification based on full wavelengths and characteristic wavelengths, respectively. Characteristic wavelengths were selected by successive projections algorithm (SPA) and competitive adaptive reweighted sampling (CARS) algorithm. SPA applies a series of vector projection operations in vector space to select a subset of variables with small collinearity and appropriate prediction ability (Liu et al. 2018). SPA has a small computational complexity, approaches the optimal selection in a short timeline, and has strong robustness (Araujo et al. 2001). CARS is a recently developed algorithm, which can select the best combination of wave numbers according to the Survival of the fittest principle in Darwin's evolution theory, providing wave number selection for establishing high-performance models. The variable selection method has been applied in multiple studies due to its fast and simple calculation, and high accuracy (Nie et al. 2016; Xu et al. 2020).

The performance of the three models was evaluated using the receiver operating characteristic (ROC) indices’ accuracy (Zweig and Campbell 1993), which calculates the ratio of correctly classified predictions to total predictions.

### Statistical analysis

ENVI version 4.7 (ITT Visual Information Solutions, Boulder, CO, USA) was employed to crop hyperspectral images. ImageJ (Version 1.54d, National Institutes of Health, Germany) was used to measure the leaf angle of rice seedlings. Python version 3.7 (Python Software Foundation) was used for spectral extraction, chemometric analysis and image visualization. Origin version 2023 (Origin Lab Corporation, Northampton, MA, USA) was utilized to prepare the graphs.

## Supplementary Information

Below is the link to the electronic supplementary material.

Supplementary file 1 (DOCX 382 kb)

## Data Availability

Datasets generated during the current study are available from the corresponding author upon reasonable request.
