# Supplementary material for: Early surveillance of rice bakanae disease using deep learning and hyperspectral imaging
Source: aBIOTECH. 2024 May 21;5(3):281–97. doi: 10.1007/s42994-024-00169-1 (PMC11399517; doi:10.1007/s42994-024-00169-1)
Supplement: Supplementary file 1 — (DOCX 382 kb) [file 42994_2024_169_MOESM1_ESM.docx]

**Supplementary material**

**Early surveillance of rice Bakanae disease using deep learning and hyperspectral imaging**

Sishi Chen^1^, Xuqi Lu^1^, Hongda Fang^2^, Anand Babu Perumal^1, 3^, Ruyue Li^4^, Lei Feng^1^, Mengcen Wang^2, 5^, Yufei Liu^1,*^

^1^ College of Biosystems Engineering and Food Science, Zhejiang University, Hangzhou 310058, China

^2^ State Key Laboratory of Rice Biology and Breeding, Zhejiang University, Hangzhou 310058, China

^3^ Computational Modeling and Nanoscale Processing Unit, National Institute of Food Technology, Entrepreneurship and Management - Thanjavur, Ministry of Food Processing Industries, Thanjavur, 613005, India

^4^ College of Environmental & Resource Sciences, Zhejiang University, Hangzhou 310058, China

^5^ Global Education Program for AgriScience Frontiers, Graduate School of Agriculture, Hokkaido University, Sapporo, 060-8589, Japan

**Corresponding author：** Yufei Liu; E-mail: [yufeiliu@zju.edu.cn](mailto:yufeiliu@zju.edu.cn)

**Supplementary Tables**

**Table S1** 5 and 10 characteristic wavelengths for each variety extracted by SPA and CARS.

| Method | Variety | Wavelength number | Wavelengths |
| --- | --- | --- | --- |
|  |  |  | (nm) |
| SPA | ZZY-8 | 5 | 558, 664, 703, 1005, 1017 |
|  |  | 10 | 553, 613, 698, 721, 817, 924, 958, 976, 994, 1017 |
|  | HZY-9326 | 5 | 553, 672, 726, 1010, 1017 |
|  |  | 10 | 533, 583, 659, 703, 724, 744, 963, 992, 1005, 1017 |
|  | ZNY-1 | 5 | 555, 664, 724, 992, 1017 |
|  |  | 10 | 495, 530, 553, 583, 657, 726, 744, 981, 1005,1017 |
| CARS | ZZY-8 | 5 | 500, 611, 669, 762, 963 |
|  |  | 10 | 451, 500, 611, 669, 687, 700, 716, 762, 835, 963 |
|  | HZY-9326 | 5 | 558, 595, 669, 752, 788 |
|  |  | 10 | 558, 575, 595, 669, 687, 700, 726, 731, 752, 788 |
|  | ZNY-1 | 5 | 578, 611, 669, 760, 976 |
|  |  | 10 | 578, 598, 611, 662, 669, 760, 895, 955, 971, 976 |

**Table S2** Classification accuracy based on characteristic wavelengths

| Day | Variety | Accuracy (%) | | | Average accuracy (%) | Parameters |
| --- | --- | --- | --- | --- | --- | --- |
|  |  | Cal | Val | Pre | Pre |  |
| 9 | ZZY-8 | 100 | 62.2 | 81.1 | 78.3 | 1500 |
|  | HZY-9326 | 98.6 | 65.2 | 65.2 |  | 1500 |
|  | ZNY-1 | 100 | 92.3 | 88.5 |  | 1500 |
| 15 | ZZY-8 | 100 | 65.0 | 80.0 | 88.1 | 1500 |
|  | HZY-9326 | 100 | 88.0 | 88.0 |  | 1500 |
|  | ZNY-1 | 100 | 76.9 | 96.2 |  | 1500 |
| 21 | ZZY-8 | 100 | 82.1 | 87.2 | 89.8 | 1500 |
|  | HZY-9326 | 92.4 | 90.9 | 86.4 |  | 1500 |
|  | ZNY-1 | 100 | 87.0 | 95.7 |  | 1500 |

Note: parameter for RBD-VGG model is epoch.

**Table S3** Number of healthy and infected rice seedling samples

| Name | Variety | The number of infected samples | The number of healthy samples (CK) |
| --- | --- | --- | --- |
| ZNY-1 | Japonica glutinous rice | 72 | 72 |
| ZZY-8 | Indica rice | 144 | 144 |
| HZY-9326 | Japonica rice | 72 | 72 |
| JHY-5 | Japonica rice | 8 | 8 |
| XS-121 | Japonica rice | 8 | 8 |

**Supplementary Figures**


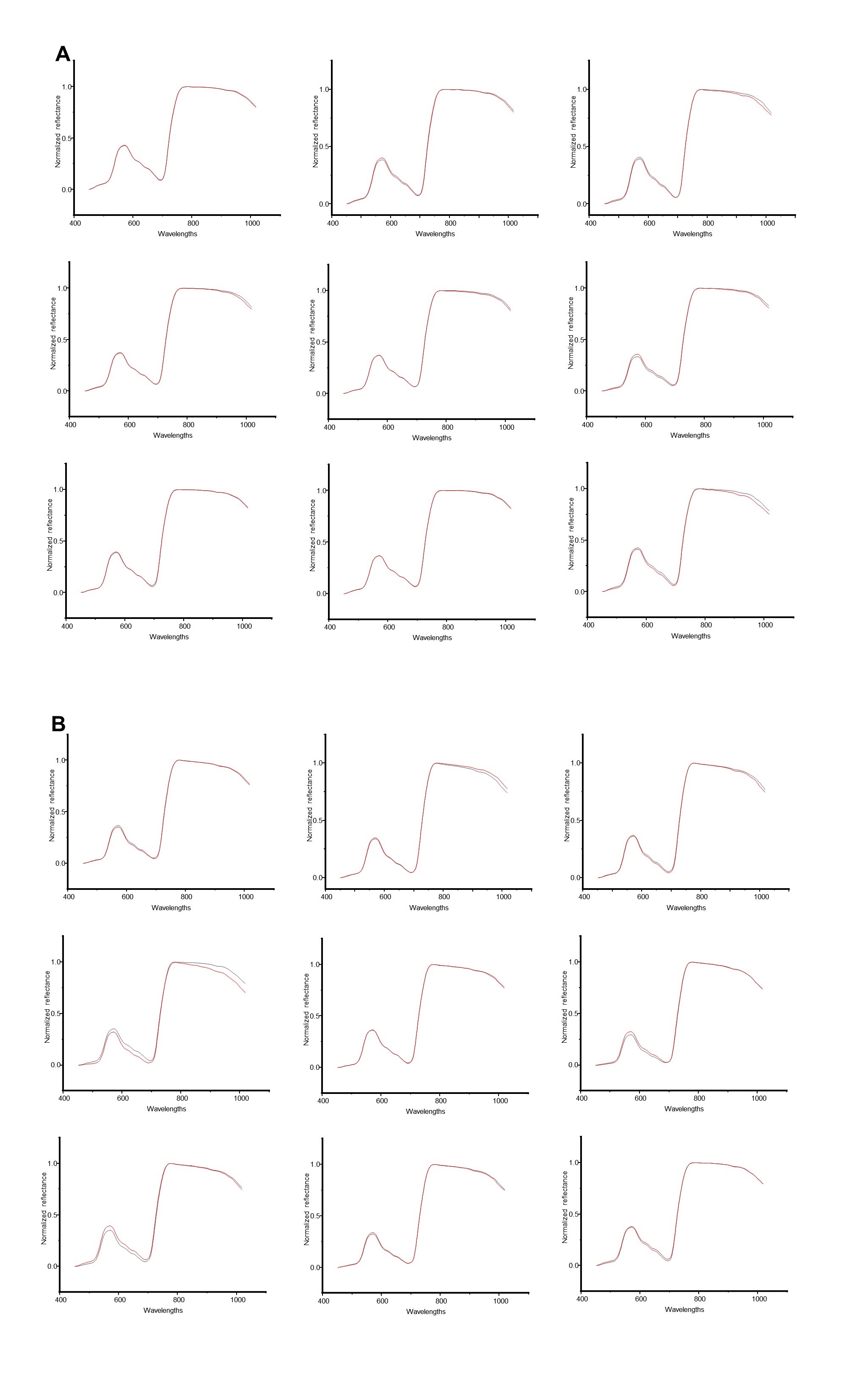


**Fig. S1** Two hyperspectral data obtained by flipping the seedling pot: (a) CK samples; (b) infected samples.
